# HCC in the Era of Emerging MASH: The Role of Ultrasound in Surveillance and New Sonographic Features in Diagnosis

**DOI:** 10.3390/cancers17244037

**Published:** 2025-12-18

**Authors:** Antonio Giorgio, Massimo De Luca, Anna Lombardi, Emanuela Ciracì, Valeria Cosima Rollo, Antonella Di Sarno, Luca Montesarchio, Giuseppe Stella, Valentina Giorgio

**Affiliations:** 1 Liver Unit, Athena Clinical Center, Piedimonte, 81016 Caserta, Italy; 2 Liver Unit, Cardarelli Hospital, 80131 Naples, Italy; marcomax@tin.it; 3 Department of Translational Medical Sciences, Federico II University Hospital, 80131 Naples, Italy; anna.lombardi@unina.it; 4 Ostuni Civic Hospital, Internal Medicine, Ostuni, 72017 Brindisi, Italy; emanuela.ciraci@asl.brindisi.it (E.C.); valeria.rollo@asl.brindisi.it (V.C.R.); 5 Department of Endocrinology, Diabetology, Andrology and Clinical Nutrition, Federico II University Hospital, 80131 Naples, Italy; antonella.disarno@unina.it; 6 General Surgery with Minimally Invasive Oncology and High Specialization in Esophageal-Gastric Procedures, Cardarelli Hospital, 80131 Naples, Italy; luca.montesarchio@aocardarelli.it; 7 Unità Operativa Semplice Dipartimentale Spina Bifida e Uropatie Congenite, Fondazione Policlinico Universitario “A. Gemelli” IRCCS, Università Cattolica del Sacro Cuore, 00168 Rome, Italy; giuseppe.stella1@guest.policlinicogemelli.it (G.S.); valentina.giorgio@policlinicogemelli.it (V.G.)

**Keywords:** hepatocellular carcinoma (HCC), HCC surveillance, ultrasound (US), contrast-enhanced ultrasound (CEUS), metabolic dysfunction-associated steatohepatitis (MASH), artificial intelligence (AI)

## Abstract

Conventional ultrasound (US) has long been central to hepatocellular carcinoma (HCC) surveillance in cirrhotic patients, due to its low cost, wide availability, non-invasiveness, and adequate sensitivity for detecting small nodules. However, its specificity in distinguishing HCC from other lesions is limited. Contrast-enhanced ultrasound (CEUS) has significantly improved the characterization of nodules first identified on conventional US. Yet, when CEUS is performed using sulfur hexafluoride (SonoVue)—the only contrast agent available in Western countries—assessment remains restricted to a single nodule per examination, and enhanced CT or MRI is still required for full characterization and staging. In clinical settings, such as hepatology, internal medicine, infectious diseases, and surgery, CEUS offers the advantage of immediate availability, enabling rapid characterization of suspicious nodules in cirrhotic livers and facilitating timely therapeutic decisions. Although the introduction of direct-acting antivirals (DAAs) has substantially reduced HCV-related HCC, HCC incidence is increasingly driven by metabolic dysfunction-associated steatohepatitis (MASH). Evidence on surveillance strategies for MASH patients remains limited, and current EASL guidelines recommend monitoring only patients with >F2 fibrosis. Additionally, the effectiveness of US in obese or diabetic/obese populations is under ongoing investigation; abbreviated non-contrast MRI has been proposed as an alternative surveillance tool, but its adoption would entail significant economic implications for healthcare systems. HCC arising from MASH—sometimes even without cirrhosis—exhibits different sonographic and pathological features. Instead of small, hypoechoic nodules, typically seen in HCV-related cirrhosis, clinicians increasingly encounter larger or multiple lesions, often accompanied by macrovascular invasion, limiting access to curative treatments. Furthermore, typical CEUS LI-RADS patterns are less frequently observed. This review summarizes the evolving US findings in the era of MASH-related HCC and underscores the continued importance of US as the primary imaging tool in routine clinical practice.

## Simple Summary

Conventional ultrasound (US) remains an essential tool for the surveillance of hepatocellular carcinoma development in patients with cirrhosis, offering acceptable sensitivity, but limited specificity. Conversely, contrast-enhanced ultrasound (CEUS) enhances HCC nodules’ characterization, although it is not accepted for detection and staging compared to CT and MRI. In recent years, metabolic dysfunction-associated steatohepatitis (MASH) has emerged as the primary cause for the development of HCC worldwide. Apart from cirrhotic patients, current guidelines do not recommend US surveillance for patients with <F3 fibrosis, even though HCC can develop from MASH without cirrhosis, leading to altered detection and treatment options. This lack of US surveillance leads to the presentation of different (large-size lesions), uncommon, confusing (CEUS appearance of non-HCC lesions), and more severe patterns of HCC (more frequent macrovascular invasion and extension), previously unseen in post-viral chronic liver disease. Furthermore, CEUS appearances are associated with equally confusing patterns, due to the presence of steatosis, and similar challenges are observed on other dynamic modalities (CT and MRI), as well as in the variability of reporting categories observed so far in chronic post-viral diseases. Despite these challenges, updated US classification systems and recent technological advances support the continued importance of US in routine clinical practice.

## 1. Introduction

### Background

Primary liver cancer is the sixth most prevalent cancer and ranks third in global cancer deaths, with significantly greater rates in developing nations and areas experiencing socioeconomic changes [1]. Hepatocellular carcinoma (HCC), which accounts for approximately 90% of primary liver cancer, remains a significant threat to global human health [2]. It is a major contributor to cancer-related deaths, with annual fatalities expected to increase significantly in the next few decades, from 800,000 in 2020 to 1,300,000 by 2040. A key factor for this trend is the worldwide increase in fatty liver disease caused by epidemics of obesity, diabetes, and metabolic abnormalities defining metabolic syndrome. Therefore, metabolic dysfunction-associated steatotic liver disease (MASLD), formerly known as NAFLD, is becoming an increasingly significant factor in global HCC incidence, although its precise impact remains unclear [3,4]. Because early-stage HCC does not present typical clinical symptoms, most patients are diagnosed at advanced stages, when surgery is no longer feasible [5]. Therefore, early diagnosis of HCC is extremely important to improving patient prognosis, as it enables access to all curative options, such as liver transplantation, resection, or ablation [6]. Consequently, over many years, programs and practice guidelines aimed at early detection of HCC nodules in high-risk individuals—particularly cirrhotic patients—have been established, mainly for patients suffering from chronic viral liver disease [7]. To date, ultrasound (US), with or without the addition of alpha-fetoprotein (AFP), remains the preferred imaging modality for monitoring high-risk patients susceptible to developing HCC [8].

This review aims to describe the emerging sonographic features observed in recent years in the evolving era of MASH-related HCC, acknowledging that US remains the primary imaging tool routinely used by clinicians for screening and diagnosis. The description of these new US features is not intended as a mere technical presentation, but rather an attempt to illustrate and disseminate, primarily from a clinical perspective, the knowledge gained in recent years regarding this new clinical entity.

## 2. Sonographic Features of HCC as First Imaging Tool Before and After the Advent of MASLD

### 2.1. Conventional Ultrasound in the Pre-MASLD Era

For many years, conventional US has played, and continues to play, a pivotal role in the surveillance of HCC in patients with liver cirrhosis [9,10]. This is due to many reasons, including its cost-effectiveness, non-invasiveness, real-time capabilities, no risk of contrast or radiation exposure and, above all, the inherent sonographic ability to detect even small nodules larger than 1 cm [11]. However, a major limitation is its operator dependency and very low specificity in characterizing nodules. In other words, standard US alone often struggles to differentiate HCC from other liver lesions, leading to inaccurate diagnosis.

### 2.2. US Findings in Viral Chronic Liver Disease

**Figure 1:**
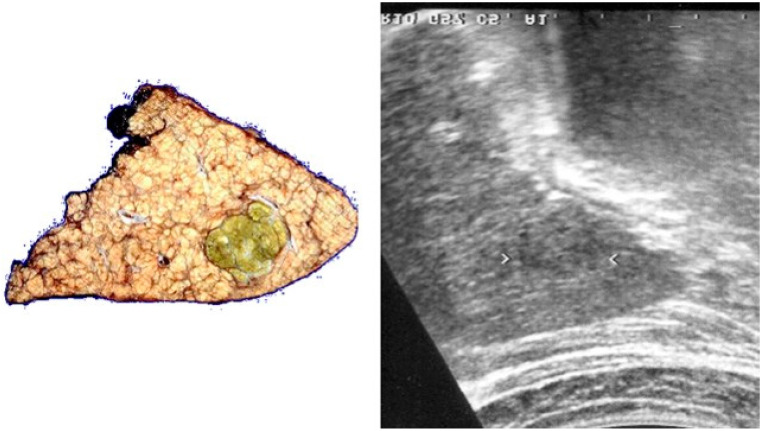
Example of very early HCC in the resected left lobe of the liver. The perfect correlation between the pathological finding (on the **left**) and the sonographic appearance of the very early HCC nodule (arrowheads) (on the **right**) is observed.

Worldwide, all major scientific associations for the study of liver diseases (the American Association for the Study of Liver Diseases [AASLD], the European Association for the Study of the Liver [EASL], the Italian Association for the Study of the Liver [AISF], the American Gastroenterological Association [AGA], the Asian Pacific Association for the Study of the Liver [APASL], the Chinese Society of Hepatology [CSH] and the Korean Association for the Study of the Liver [KASL]) recommend performing abdominal US every 6 months in chronically infected HBV patients and/or HCV-related cirrhotic patients for the early detection of HCC [12,13,14,15,16,17,18,19]. This policy aims to detect small nodules in high-risk patients, enabling optimal curative options for long-term survival, such as liver transplantation, resection, or ablation (Figure 1). In these patients, the main US characteristics of very early and early HCC are typically a small, hypoechoic lesion (i.e., a space-occupying lesion of <2 or <3 cm in diameter, respectively, with lower echogenicity than the surrounding liver parenchyma) and the absence of macrovascular invasion [20].

In cases of advanced HCC, US demonstrates great capability in detecting macrovascular invasion in the main portal vein and its branches.

**Figure 2:**
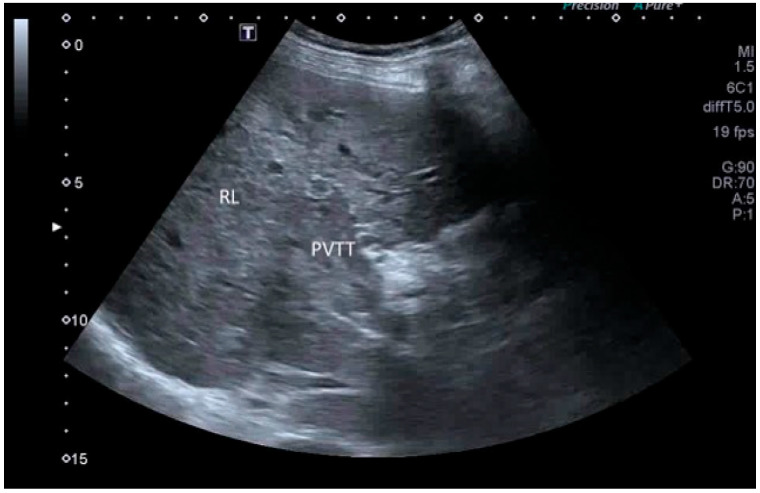
PVTT on conventional US. The main portal trunk is filled with solid material similar to the surrounding liver. PVTT, portal vein tumor thrombus; RL, right lobe of the liver.

US shows high sensitivity and high specificity in the diagnosis of malignant tumor thrombus in the main portal vein (PVTT) and its branches and/or hepatic veins, or even in the inferior vena cava or right atrium, showing the presence of solid material in these vessels that resembles the HCC US echotexture, often associated with the enlargement of the main portal trunk (Figure 2).

### 2.3. New, Different Sonography Features in the MASLD Era

Regarding the sonographic appearance of HCC in the MASLD era, important findings have emerged when nodules are detected in patients with MASLD-related cirrhosis. Notably, these features are also observed in MASLD patients without cirrhosis, reflecting the possibility of direct progression from steatohepatitis to HCC.

**Figure 3:**
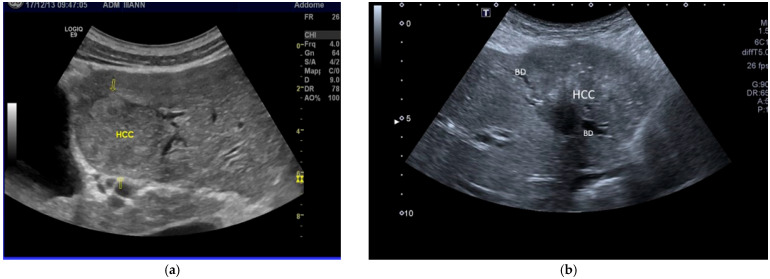

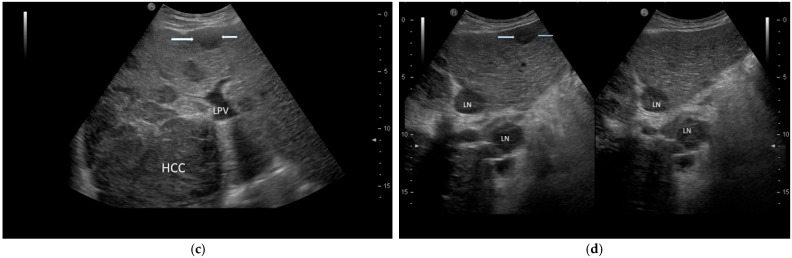
(**a**). Large HCC (arrow) in MASLD-related cirrhosis with infiltration of portal bifurcation between 2nd and 3rd segments of the liver. (**b**). MASLD HCC at first presentation in patients without cirrhosis. A large, inhomogeneous mass is present in the left lobe of the liver, accompanied by biliary invasion. BD, dilated biliary duct. (**c**). MASLD HCC. A large hypoechoic mass with irregular borders is present in the 7th segment of the liver, accompanied by other hypoechoic, small nodules in the 4th segment (arrows). LPV, left portal vein. (**d**). The multinodular HCC in 6c is accompanied by metastatic enlarged mesenteric lymph nodes (LNs).

Piscaglia and colleagues were the first to describe the clinical and sonographic changes now observed in HCC in NAFLD (currently referred to as MASLD) [21]. The authors compared the characteristics of HCV-related HCC with those observed in NAFLD–HCC. They found that, compared to HCC associated with HCV chronic infection, NAFLD–HCC nodules are larger and often accompanied by macrovascular or biliary invasion (Figure 3a–d). Furthermore, these large nodules were observed in patients not under US surveillance (see later), and only 50% of NAFLD–HCC patients were cirrhotic.

**Figure 4:**
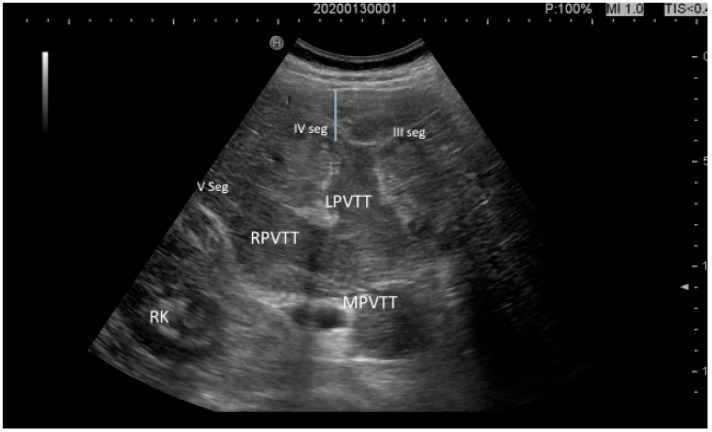
Extensive macrovascular invasions in a non-cirrhotic MASLD patient, involving the main portal vein trunk (MPVTT), the right branch of the portal vein (RPVTT), the left branch of the portal vein (LPVTT), and segmental branches for the 3rd, 4th, and 5th segments; RK, right kidney.

Dong and colleagues conducted a multicenter study reporting conventional US findings in 96 patients with histologically proven HCC without cirrhosis. They confirmed the appearance of large-sized HCC nodules (up to 9 cm in diameter) in 62 out of 96 patients (64.5%) with a heterogeneous US pattern (neither small nor hypoechoic, as typically observed in post-viral forms) and with irregular margins (Figure 4). None of these patients was under surveillance for HCC [22].

Table 1 summarizes the different sonographic patterns in post-viral and MASLD-related HCC.

## 3. Contrast-Enhanced Ultrasound

The possibility to study ultrasound contrast agent (UCA) microbubbles non-invasively, rapidly, safely, and without radiation exposure of the vascular supply of focal liver lesions enables the characterization of space-occupying hepatic lesions, facilitating the differentiation of benign from malignant lesions, and their definition, with high specificity [23].

Therefore, the clinical use of UCA microbubbles has revolutionized the study of focal liver lesions in hepatology [23].

Currently, two main US contrast agents are available on the market. The first, SonoVue, is composed of sulfur hexafluoride. It was the first low-mechanical index UCA with no destruction of microbubbles and is a pure blood stream UCA. It is internationally distributed across Western and Asian countries, with FDA approval in the USA [24]. The second, Sonazoid, is available in Japan, South Korea, Denmark, Norway, and recently in China [25]. Unlike SonoVue, Sonazoid’s gas-filled microbubbles provide the benefit of enabling highly stable Kupffer phase imaging for up to 60 min, and vascular phases. Thereafter, Sonazoid is phagocytosed by Kupffer cells. Since HCC nodules lack Kupffer cells, they appear as hypovascular nodules on US at 8–10 min according to WFUMB. These properties make Sonazoid a useful tool for contrast-enhanced detection of HCC nodules (see later) [26].

**Figure 5:**
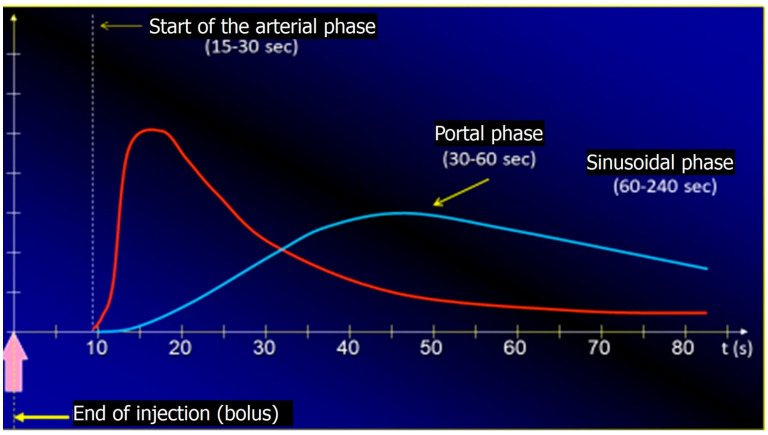
Schematic representation of arterial, portal, and sinusoidal phases after SonoVue injection.

Figure 5 shows the dynamic curves with timing of the arterial (red), portal (blue) and sinusoidal (blue) phases occurring after IV SonoVue injections.

**Figure 6:**
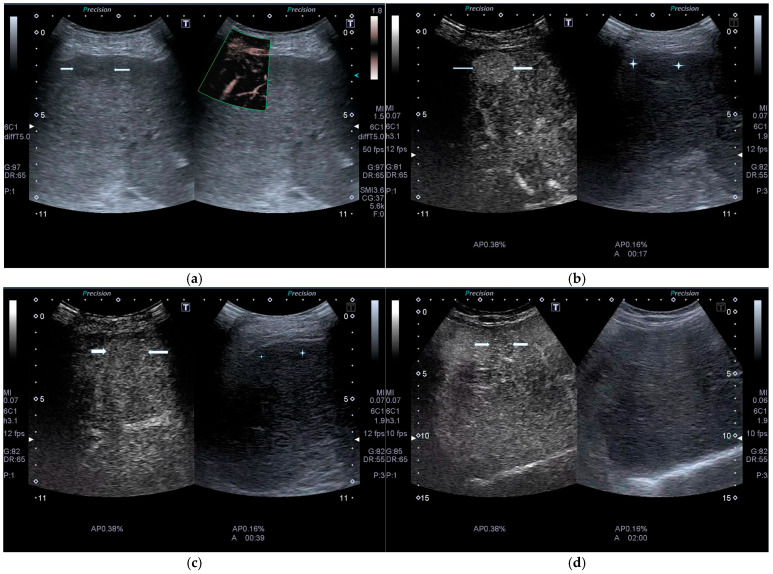
(**a**). CEUS of early HCC. Left: conventional US shows a small, hypoechoic, subcapsular nodule (arrows) in the 6th segment of the liver. Right: micro flow imaging (MFI). (**b**). The HCC nodule shows rapid (8 s after SonoVue injection), homogeneous, non-rim, non-globular, hyperechoic enhancement (left arrows). On the right is a comparative conventional US image (stars). (**c**). At 50 s post-injection, the HCC nodule (arrows) appears isovascular. (**d**). At 120 s post-injection, the HCC nodule (arrows) shows mild wash-out.

Consequently, CEUS (similar to enhanced Computed Tomography [eCT] and enhanced Magnetic Resonance Imaging [eMRI]) allows for the visualization of homogeneous hyperenhancement of HCC nodules in the arterial phase, lasting 10–30 s after IV UCA injection (the nodule appears hyperechoic during UCA wash-in). During the portal phase (30–60 s), contrast begins to be released (wash-out), and by the late portal phase (>120 s), the UCA is eliminated and the nodule appears hypovascular (hypoechoic) (Figure 6a–d) [24].

Accordingly, CEUS is suggested as a primary diagnostic technique for focal liver lesions. The sensitivity of CEUS in diagnosing hepatic nodules was reported to exceed 95%, with specificity nearing 90% [27,28,29,30].

In a review by Westwood et al., CEUS using SonoVue showed similar diagnostic value to eCT and eMRI. Furthermore, CEUS is more cost-effective than eMRI [31].

The clinical value of using CEUS-SonoVue for HCC diagnosis has been endorsed by the AISF, APASL, and the Chinese and Korean Associations. Conversely, AASLD and EASL do not yet recognize the diagnostic capability of this UCA [32,33]. When CEUS is performed using SonoVue, its main limitation is the ability to assess only one nodule, or, at most, two nodules in the same plane, at a time [30]. Nevertheless, in routine clinical practice, when a newly detected or recurrent nodule is identified in a cirrhotic patient, CEUS enables a rapid diagnostic and therapeutic work-up in hepatology, internal medicine, infectious diseases, or surgery.

## 4. CEUS-LI-RADS Category Classification in Non-MASLD HCC

As occurred with the introduction of LI-RADS for eCT and eMRI in 2017, the American College of Radiology (ACR) introduced CEUS to standardize reporting and provide a universal interpretative framework for the various universal HCC patterns observed in patients at high risk for HCC [34]. The ACR introduced five categories, ranging from 1, “definitively benign”, to 5, “definitively HCC”. CEUS LI-RADS 5 is characterized by the presence of homogenous, non-rim, non-globular hyperenhancement in the arterial phase, followed by mild and late wash-out (>60 s) in the portal and sinusoidal phases [35]. Table 2 describes the CEUS LI-RADS classifications, along with characteristics and management.

The presence of mild and, in particular, more than 60 s wash-out, is crucial, as it enables the differentiation of HCC from non-HCC malignancies, such as metastases, neuroendocrine tumors (NETs), hepatic lymphoma, and especially intrahepatic cholangiocarcinoma, which has been increasing in incidence in recent years in liver cirrhosis.

CEUS-LI-RADS classification has demonstrated high sensitivity and a very high specificity in diagnosing HCC. In our study, the latter reached 100% when the arterial hyperenhancement was used as a hallmark, even if intrahepatic cholangiocarcinoma (ICC) nodules were not encountered in our cases [34]. Other authors have similarly confirmed the high specificity for CEUS LI-RADS 5 category. Terzi et al. retrospectively analyzed 848 high-risk patients for HCC with 1006 nodules from five centers in Italy [36] and reported a rate of correct HCC diagnosis of 99% (515 of 519, LR-5) based on category 5 classification.

### CEUS Findings in MASLD Cirrhotic and Non-Cirrhotic HCC

**Figure 7:**
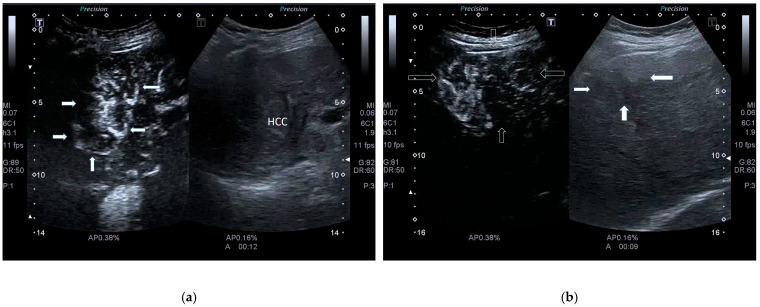
(**a**). CEUS in HCC MASLD cirrhotic patient. In arterial phase, inhomogeneous arterial enhancement is present. (**b**). CEUS of large HCC in non-cirrhotic MASH patient. In arterial phase, the nodule shows heterogeneous hyperenhancement. Note that, on CEUS, the HCC nodule is larger compared to conventional US on the right (full arrows).

**Figure 8:**
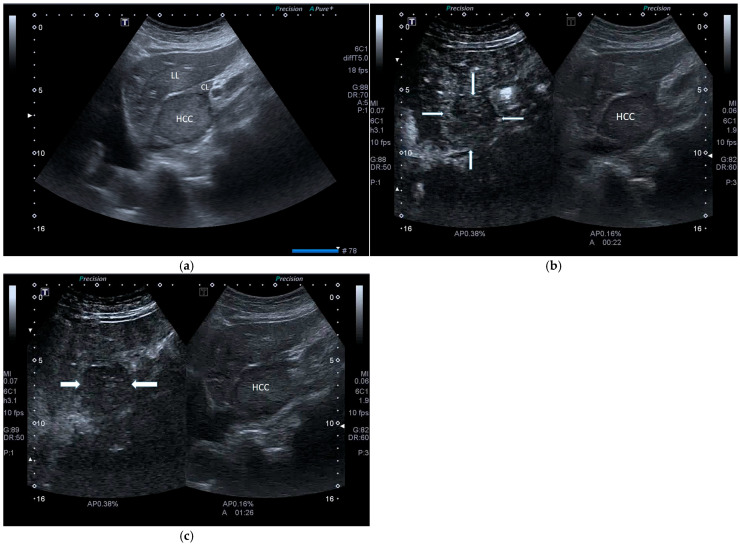
(**a**). Conventional US of a large HCC of the caudate lobe (CL). LL, left lobe. (**b**). On SonoVue CEUS, at 20 s, the nodule presents rapid wash-out (arrows on the left image). (**c**). On SonoVue CEUS, at 35 s, the wash-out is already completed (arrows on the left image).

Previous CEUS post-viral HCC features change when HCC arises in MASLD patients. Uncommon and sometimes confusing CEUS findings have been reported in the literature in cirrhotic and non-cirrhotic MASLD patients. Dong and colleagues reported that, in non-cirrhotic livers examined with SonoVue, 78.1% (75/96) of HCC nodules showed heterogeneous hyperenhancement compared with the surrounding liver parenchyma during the arterial phase, instead of homogeneous hyperenhancement, typically seen in post-viral chronic liver disease (Figure 7a,b). Moreover, surprisingly, 30% of HCC lesions showed early wash-out (<60 s) (Figure 8a–c). As mentioned previously, such features resemble those of non-HCC malignant lesions, such as liver metastases, neuroendocrine tumors (NETs), and, mainly, ICC. In fact, according to CEUS LI-RADS, these features are characterized as CEUS LI-RDS M, i.e., non-malignant HCC [22,37].

In a more recent study by the same authors, using Sonazoid in NAFLD-related HCC, the CEUS findings were not consistent with characteristics of CEUS LI-RADS categories. Dong and colleagues compared Sonazoid findings between HBV-related HCC and NAFLD-related HCC. In approximately half of NAFLD-related HCCs, HCC nodules showed an early wash-out (<60 s)—the exact opposite of the classic post-viral HCC nodule. In Dong’s study, post-viral HCC nodules showed the expected slow and delayed wash-out pattern, consistent with the established CEUS LI-RADS categories [38]. This is extremely important in the clinical setting, since, as mentioned, a liver biopsy is essential to characterize the nodule. Consequently, the dynamic imaging (LI-RADS 5) typical of post-viral HCC—which rendered histological analysis redundant and resulted directly in ablation, resection, and potentially transplantation—will no longer be possible to follow.

On Sonazoid-CEUS, more than half of the NAFLD-related HCCs exhibited relatively early and mild wash-out within 60 s (54.2%, 39/72), whereas most HBV cirrhosis-related HCCs displayed wash-out between 60 and 120 s (46.8%, 37/79), or after 120 s (39.2%, 31/79), with a statistically significant difference (*p* < 0.001) [38].

**Figure 9:**
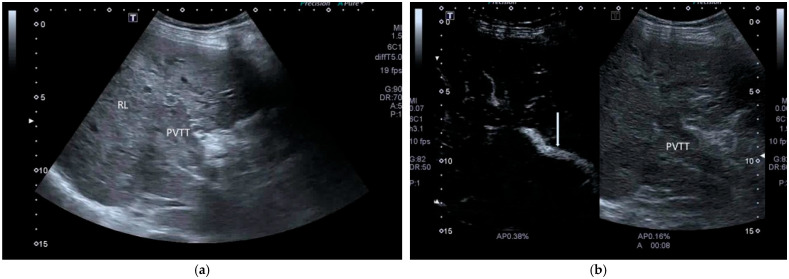

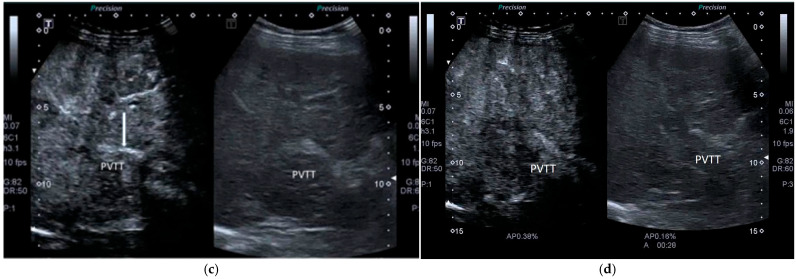
(**a**). PVTT, portal vein tumor thrombus; RL, right liver. (**b**). SonoVue CEUS. The arterial phase at 8 s clearly shows the hepatic artery as a hyperechoic serpiginous vessel (arrow). PVTT, portal vein tumor thrombus. (**c**). At 18 s, the PVTT shows uniform hyperenhancement, suggesting the presence of cancer. The hepatic artery remains identifiable (arrow). (**d**). At 130 s, the PVTT presents wash-out.

The same diagnostic approach can be applied to the assessment of HCC with portal vein tumor thrombus (PVTT). CEUS can demonstrate arterial hyperenhancement, followed by late and mild wash-out within the thrombus in the PV—features that are pathognomonic for tumor thrombus (Figure 9a–d). In contrast, benign PVTT is characterized by the absence of arterial enhancement. CEUS demonstrates high sensitivity (90.9%) and 100% specificity in differential diagnosis of these two conditions [39].

In contrast, in NAFLD–HCC patients, CEUS is characterized by lower sensitivity and specificity compared to post-viral cirrhosis, probably due to the low-mechanical index setting used during CEUS examination [40,41].

However, CEUS sensitivity and specificity may also decrease when CEUS–Sonazoid is used under suboptimal imaging conditions. Putz JF et al. found that, in obese patients (who represent the majority of MASLD patients), Sonazoid diagnostic accuracy decreased from 98% in patients with good acoustic conditions to 92.6% [16,42].

Table 3 summarizes the CEUS findings observed in post-viral and MASLD-related HCC.

## 5. US Surveillance of HCC: Old Policies and New Alternative Features Added for the Rising MASH Era

To align US with LI-RADS (eCT, eMRI) and CEUS LI-RADS, the American College of Radiology (ACR) introduced the US Liver Imaging Reporting and Data System (US LI-RADS), in 2017, for the surveillance of patients at risk of HCC. This initiative aimed to standardize the US examination among all physicians involved in US surveillance of HCC, and to homogenize reporting, thereby improving patient management. The 2017 version was updated in 2024 to incorporate new scientific evidence accumulated over the past seven years, and is now referred to as LI-RADS US Surveillance version 2024 [43]. Importantly, this updated US categorization has been aligned with the 2023 American Association for the Study of Liver Diseases (AASLD) Practice Guidelines on HCC management [12]. Table 4 describes the US LI-RADS categories, along with their characteristics and management.

### US Comparison Between CT and MRI in Surveillance of MASH-Related HCC

A recent study by Lima et al. suggested that primary surveillance strategies using CT or MRI may be more cost-effective than US-based programs, due to increased sensitivity and specificity for disease detection [44]. However, the study did not consider the economic impacts of eMRI false-positive results or radiation exposure from repeated eCT scans (that can be repeated every three or six months in the surveillance program as US) [45,46]. Moreover, issues such as claustrophobia, contrast injection intolerance, and limited MRI accessibility make CT/MRI-based surveillance impractical in many healthcare systems, even in countries with universal coverage (e.g., Italy) [47]. Patient adherence to US-based surveillance already remains low (18–25%) [47], and such factors could further reduce compliance. Furthermore, US LI-RADS categories and VISs were primarily designed for patients with post-viral or alcohol-related chronic liver disease. Current guidelines do not recommend HCC screening in MASLD patients without advanced fibrosis.

Specifically, AASLD guidelines advise against surveillance in non-cirrhotic MASLD patients [12], the EASL recommends considering patients with F3 fibrosis based on individual risk [13], and the AGA supports screening in those with advanced fibrosis or cirrhosis [48].

Cost-effectiveness analyses suggest that surveillance is justified in populations with an HCC incidence ≥ 1% per year [49], which is higher than current rates in non-cirrhotic MASLD patients. Additionally, studies have shown that US evaluation can be limited in MASLD, potentially missing up to 41% of HCC cases, with significantly lower sensitivity compared to other etiologies of chronic liver disease [50]. Factors such as high BMI, thicker subcutaneous fat layer, and severe steatosis contribute to beam attenuation, frequently resulting in a VIS-C score. Nevertheless, analysis based on ITA.LI.CA HCC database demonstrates that MASLD-HCC nodules are often larger and more metastatic at the diagnosis, with less susceptibility to curative therapies compared to non-MASLD-HCC [51]. This underscores the critical role of early detection in improving prognosis of MASLD-HCC patients.

The debate regarding alternatives to US surveillance of HCC remains ongoing [52]. US LI-RADS appropriately highlights when US examinations are technically inadequate and when to switch to alternative modalities in patients with obesity or other limitations. However, the optimal alternative imaging method remains unclear. Notably, most evidence supporting the superiority of MRI over ultrasound for HCC surveillance derives from Eastern populations, composed predominantly of patients with chronic HBV infection. A recent multicenter retrospective study in the United Kingdom by Spiers et al. included 1780 patients with post-viral chronic liver disease, alcohol-related liver disease (ArLD), and NAFLD, as well as non-cirrhotic patients screened with US. The study demonstrated that survival was significantly associated with regular HCC surveillance in post-viral chronic liver disease, whereas no survival benefit was observed among patients with ArLD or NAFLD [53]. Conversely, El Sabagh et al. conducted a large retrospective study including 1954 patients undergoing strict surveillance with CT, MRI, and US. Their findings showed no significant differences in clinical outcomes according to the imaging modality used for surveillance, provided that surveillance was performed rigorously, and patient compliance was optimal [54]. Finally, the earlier research by Park et al. evaluated the use of Sonazoid contrast-enhanced ultrasound (CEUS)—owing to its ability to visualize the Kupffer phase—for the detection of HCC nodules during surveillance [55,56]. Comparing conventional US with Sonazoid CEUS, the study found no significant advantage of Sonazoid over standard US. Despite the study’s limitations, its findings tempered previous expectations regarding the potential of CEUS to enhance HCC detection and staging, at least in the liver. These results suggest that CEUS, using either sulfur hexafluoride or perfluorobutane microbubbles, remains primarily a tool for the characterization of HCC in cirrhotic livers, rather than for surveillance. Conversely, conventional US demonstrated satisfactory sensitivity (75.0%) and accuracy (95.6%), supporting its use in surveillance programs. While CEUS offers limited benefit for HCC surveillance, it substantially improves the detection of liver metastases, increasing sensitivity by up to 40% when added to standard US [57].

## 6. AI and MASLD Surveillance and Diagnosis

Artificial intelligence (AI) has been rapidly employed in the diagnosis of diffuse liver disease, staging of liver steatosis, fibrosis, and the detection of HCC [58]. Numerous studies emphasized the capability of AI in defining the fibrosis stage in chronic liver disease and in diagnosing HCC using US through deep learning, machine learning, and big data approaches, with good performance [59]. In particular, Dana and colleagues employed two deep learning models called “STARHE-RISK” and “STARHE-DETECT”, which integrate US cine-loop images with scores based on clinical and laboratory parameters; these models showed good accuracy in risk-stratification and detection of early HCC, respectively [60]. Furthermore, according to the authors, both AI models are user-friendly for radiologists and physicians. In conclusion, the authors proposed that such models could serve as a reliable surveillance tool for HCC, potentially paving the way for a risk-based, personalized surveillance program that integrates US imaging with patient-specific clinical and laboratory data. Moreover, CEUS may further enhance US capability in the differential diagnosis of focal liver lesion [FLLs] [61]. Integrating AI into US or CEUS would overcome one of the main limitations of US—its high operator dependency—which is particularly critical in the assessment of chronic liver disease by less-experienced physicians. Lupsor-Platon et al. specifically reviewed the contribution of AI in MASLD-related HCC diagnosis, highlighting future perspectives and potential goals, although specific features of sensitivity and specificity data were not reported [62]. A very recent, interesting study by Nakatsuka et al. presented AI data on prediction of HCC in MASLD patients using a deep learning model based on simple hematoxylin and eosin whole-slide images of biopsy-proven steatotic liver disease. The authors concluded that this model could recognize the initial pathological changes from mild fibrosis to progression towards overt HCC in MASLD patients with steatotic liver disease [63]. Despite these promising advances, to our knowledge, no study has yet directly addressed the use of AI for the specific diagnosis of HCC in MASLD patients. Future AI research on HCC surveillance in MASH patients should focus on prospective, multicenter, and potentially validated global clinical trials. Additionally, development of multimodal AI models that integrate imaging with clinical, genomic, and transcriptomic information may further improve risk assessment, facilitate early HCC diagnosis, and personalize treatment strategies.

## 7. Conclusions

Currently, US represents the first-line imaging modality worldwide for HCC surveillance in patients with liver cirrhosis, with broad consensus; however, differences exist among European, American, and Asian-Pacific scientific societies. In our opinion, the problem concerns the timing and target population for surveillance. It is objectively difficult to recommend surveillance for a patient with isolated US evidence of fatty liver disease, without elevated aminotransferases, and the appropriate surveillance interval remains uncertain. Moreover, MASH may coexist with normal aminotransferases, raising the question of whether surveillance should instead be guided by transient elastography or, ideally, US shear wave elastography. The latter, being incorporated into the ultrasound machine, could be a useful solution, allowing for the immediate measurement of both fibrosis and steatosis simultaneously with the visualization of a bright liver. When a new or recurrent nodule is detected during US surveillance in a cirrhotic patient, it should always be considered HCC until proven otherwise. Nonetheless, if US fails in recognizing early HCC nodules in MASLD patients, abbreviated MRI represents an alternative to overcome such failure, as shown in recent years. Nevertheless, its implementation must take into account differences in healthcare systems, whether private or universal, as well as patient adherence, claustrophobia, and waiting times—all of which may limit its widespread adoption. Advances in AI-integrated US and CEUS technologies hold promise for overcoming these challenges. AI-assisted ultrasound, already available in next-generation US machines, is likely to enhance lesion detection, reduce operator dependency, and improve the overall accuracy and consistency of HCC surveillance.

## Figures and Tables

**Table 1:** Differences between conventional sonographic features of post-viral and MASLD HCC.

POST-VIRAL HCC	MASLD HCC
(1) Small, hypoechoic, well-defined nodules identified during surveillance	(1) Large, inhomogeneous nodules up to 8–9 cm in diameter with irregular borders
(2) Low presence of macrovascular invasion	(2) Accidental discovery
(3) All therapeutic options are available (transplantation, resection, ablation)	(3) High prevalence of macrovascular invasion or metastatic sites
	(4) Limited chance of transplantation if only a single resection is performed or in absence of cirrhosis
	(5) Consider conversion therapy if vascular invasion is present

**Table 2:** CEUS LI-RADS classifications (SonoVue).

CEUS LI-RADS CLASS	FEATURES	MANAGEMENT
CEUS LR-1 (definitely benign)	- cyst; hemangioma; - hepatic fat deposition/sparing	return to 6-monthly surveillance
CEUS LR-2 (probably benign)	- distinct isoenhancing solid nodule < 10 mm; if ≥10 mm, then apply CEUS LR-3 - CEUS LR-3 nodules with size stability for ≥2 years	return to 6-monthly surveillance
CEUS LR-3 (intermediate malignancy probability)	- nodule < 20 mm no APHE and no wash-out, or with late and mild wash-out; - nodule ≥ 20 mm no APHE and no wash-out; - nodule < 10 mm with APHE and no wash-out	- assess with alternative diagnostic imaging (CT or MRI) in ≤6 months; - repeat CEUS in ≤6 months - consider multi-disciplinary discussion
CEUS LR-4 (probably HCC)	- nodule ≥ 20 mm no APHE, with late and mild wash-out; - nodule < 10 mm with APHE, with late and mild wash-out; - nodule ≥ 10 mm with APHE, and no wash-out	- consider multi-disciplinary discussion - if biopsy/treatment is not planned, repeat assessment with CEUS or alternative imaging in ≤3 months
CEUS LR-5 (definitely HCC)	- nodule ≥ 10 mm with APHE, and with late and mild wash-out	- multi-disciplinary discussion for consensus management
CEUS LR-M (probably or definitely malignant, but not HCC-specific)	- rim (non-peripheral discontinuous globular) APHE, or early (<60 s) wash-out, or marked wash-out	- multi-disciplinary discussion for consensus management
CEUS LR-TIV (tumor in vein)	- unequivocal enhancing tissue in vein, regardless of a parenchymal mass	- multi-disciplinary discussion for consensus management
CEUS LR-NC (cannot be categorized)	- due to image degradation or omission	- assess with alternative diagnostic imaging (CT or MRI) in ≤6 months; - repeat CEUS in ≤6 months; - consider multi-disciplinary discussion

APHE—arterial phase hyperenhancement.

**Table 3:** CEUS differences between post-viral and MASLD HCC (SonoVue).

POST-VIRAL HCC	MASLD HCC
(1) Rapid homogeneous hyperenhancement (wash-in UCA) in the arterial phase	(1) Inhomogeneous hyperenhancement in the arterial phase (75–90% of cases).
(2) Mild wash-out in the portal and sinusoidal phases (>60 s)	(2) Intense wash-out (<60 s).
(3) Late wash-out in the portal and sinusoidal phases (>60 s)	(3) Rapid wash-out (<60 s).
	These characteristics would be focused more on the lack of malignancy rather than HCC.
	Need for liver biopsy to make the diagnosis.

**Table 4:** LI-RADS US HCC Surveillance (version 2024).

US Categories	FEATURES	MANAGEMENT
US-1, Negative (definitely benign)	No nodule suspicious for HCC is observed in the liver (e.g., simple cyst, hemangiomas, focal fatty sparing pericholecystic)	Continue 6-month interval US surveillance
US-2, Sub-threshold (not definitely benign)	At least one nodule < 10 mm	Repeat US every 3–6 months (low probability of these nodules progressing to HCC in a cirrhotic liver)
US-3, Positive (advanced HCC)	Detection of at least one or more nodules > 10 mm	CEUS, eCT, or eMR must be performed

## Data Availability

No new data were created or analyzed in this study. Data sharing is not applicable to this article.
